# A Novel Brain–Computer Interface Virtual Environment for Neurofeedback During Functional MRI

**DOI:** 10.3389/fnins.2020.593854

**Published:** 2021-01-11

**Authors:** Halim I. Baqapuri, Linda D. Roes, Mikhail Zvyagintsev, Souad Ramadan, Micha Keller, Erik Roecher, Jana Zweerings, Martin Klasen, Ruben C. Gur, Klaus Mathiak

**Affiliations:** ^1^Department of Psychiatry, Psychotherapy and Psychosomatics, Medical School, RWTH Aachen University, Aachen, Germany; ^2^Jülich Aachen Research Alliance-Translational Brain Medicine, RWTH Aachen University, Aachen, Germany; ^3^Department of Psychiatry, Perelman School of Medicine, University of Pennsylvania, Philadelphia, PA, United States

**Keywords:** neurofeedback (NF), brain–computer interface, self-regulation, methodology development, virtual environment (VE), real-time fMRI (rtfMRI)

## Abstract

Virtual environments (VEs), in the recent years, have become more prevalent in neuroscience. These VEs can offer great flexibility, replicability, and control over the presented stimuli in an immersive setting. With recent developments, it has become feasible to achieve higher-quality visuals and VEs at a reasonable investment. Our aim in this project was to develop and implement a novel real-time functional magnetic resonance imaging (rt-fMRI)–based neurofeedback (NF) training paradigm, taking into account new technological advances that allow us to integrate complex stimuli into a visually updated and engaging VE. We built upon and developed a first-person shooter in which the dynamic change of the VE was the feedback variable in the brain–computer interface (BCI). We designed a study to assess the feasibility of the BCI in creating an immersive VE for NF training. In a randomized single-blinded fMRI-based NF-training session, 24 participants were randomly allocated into one of two groups: active and reduced contingency NF. All participants completed three runs of the shooter-game VE lasting 10 min each. Brain activity in a supplementary motor area region of interest regulated the possible movement speed of the player’s avatar and thus increased the reward probability. The gaming performance revealed that the participants were able to actively engage in game tasks and improve across sessions. All 24 participants reported being able to successfully employ NF strategies during the training while performing in-game tasks with significantly higher perceived NF control ratings in the NF group. Spectral analysis showed significant differential effects on brain activity between the groups. Connectivity analysis revealed significant differences, showing a lowered connectivity in the NF group compared to the reduced contingency-NF group. The self-assessment manikin ratings showed an increase in arousal in both groups but failed significance. Arousal has been linked to presence, or feelings of immersion, supporting the VE’s objective. Long paradigms, such as NF in MRI settings, can lead to mental fatigue; therefore, VEs can help overcome such limitations. The rewarding achievements from gaming targets can lead to implicit learning of self-regulation and may broaden the scope of NF applications.

## Introduction

After the commercial availability of video games in the 1970s, they have become ubiquitous and part of everyday life. Nowadays, video gaming is commonplace in more than 65% of the American population and is one of the major forms of entertainment (ESA, 2019). The technical development has made available virtual environments (VEs) that simulate a variety of behaviors. Examples include, but are not limited to, reinforcing positive behaviors (Kuo et al., 2016), improving teaching skills (Kleinert et al., 2015), monitoring of everyday behaviors (Pouke and Häkkilä, 2013), analyzing sports performance (Bideau et al., 2010), and studying aggressive behaviors (Weber et al., 2006; Jeong et al., 2012). Several studies assessed gaming behavior and how it translates into everyday behavior (Granic et al., 2014). Such an effect of gaming on quotidian behavior can be used as a training model. In combination with a brain–computer interface (BCI), closed-loop reinforcement and training of brain activity can be achieved. Using real-time functional magnetic resonance imaging (rt-fMRI) with a BCI, specific localized brain areas can be targeted for self-regulation. This method can address disorders characterized by altered neural activation patterns (Ruiz et al., 2013; Scharnowski and Weiskopf, 2015; Zweerings et al., 2018). This article describes the development of a VE-BCI for the self-regulation of localized brain activity during rt-fMRI.

### Gaming Environments in Research

As gaming has become a significant part of our private, social, and professional activities, it has been the subject of many academic and scientific studies assessing the long- and short-term effects of gaming. Different aspects of everyday life, exhibited shortcomings, and benefits of video games such as socializing (Dalisay et al., 2015; Carras et al., 2017), learning ability (Vogel et al., 2006), motivation for activities (Ventura et al., 2013), drug-attenuating behavior (Klasen et al., 2013), emotion regulation (Wolf et al., 2018), and behavioral modification (Behm-Morawitz et al., 2016) have been assessed. Playing video games can have a positive influence on cognitive, motivational, emotional, and social functioning. For instance, reward system responses during video games, specifically immersive puzzle games, may facilitate flexible learning, reinforcing adaptive strategies, and inhibiting frustration from failure to apply reappraisal strategies (Granic et al., 2014). Similarly, playing video games, specifically action games that often comprise violent content, may improve cognitive functions such as short-term memory, spatial cognition, decision-making, and reaction times when comparing video game players to non–video game players (Bavelier et al., 2012).

### Neuroscientific Tasks in Gaming Environments

Video games and VE software platforms are increasingly used in neuroscience over the recent years. Such software platforms provide flexibility in modifying and controlling the VE to a great degree, especially compared to traditional stimuli such as videos, pictures, and text (Bailenson et al., 2003; Courtney et al., 2010; Kozlov and Johansen, 2010). In a VE, multimodal stimuli can be generated and precisely controlled. With recent technological developments and implementations becoming free for the public to use, there is the possibility to achieve high visual and auditory fidelity at a reasonable investment. Such environments can create analogs of any physical environment and engage the participants, offering a high level of immersion (Blascovich et al., 2002; Garau et al., 2003). In the context of experimental paradigms, VEs enable investigations of behavior and social interactions in scenarios with a high ecological validity. The virtual interactions elicit consistent brain responses to seminaturalistic behaviors (Mathiak and Weber, 2006; Klasen et al., 2012). Furthermore, the VE platform offers replicability and reproducibility of the experiments (Parsons et al., 2013).

Immersive VEs have been employed to target and reduce psychopathological symptoms in depression patients using therapeutic self-compassion via self-embodiment with virtual avatars (Falconer et al., 2016). VEs have also been shown to provide a unique platform for online support groups. In such a setting, VEs provide a meaningful interaction between participants to engage with and has shown to reduce subjective perceived stress (O’Connor et al., 2014).

### Neurofeedback Modalities

**FIGURE 1:**
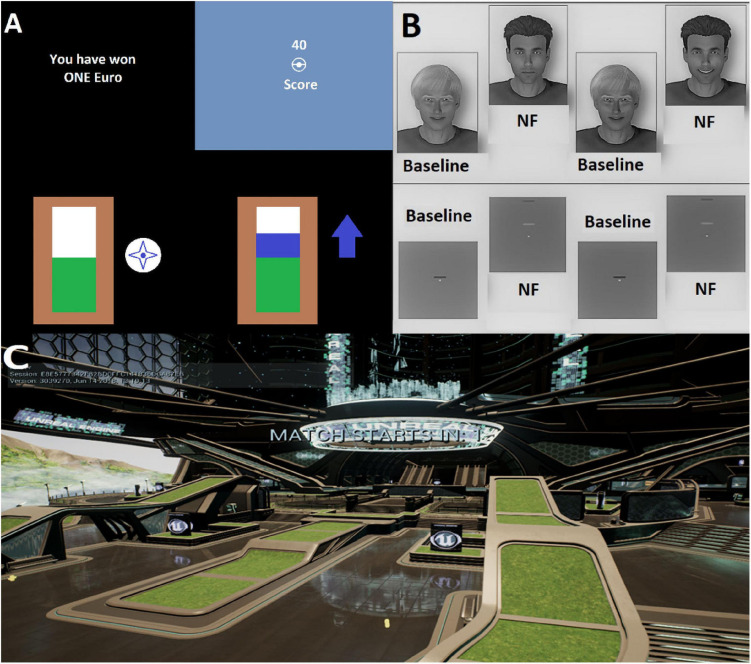
**(A)** An overview of current methods used as NF modalities (Bray et al., 2007; Rota et al., 2009; Ruiz et al., 2013). These images have been modified and reimagined from the original and grouped together to concisely show the general NF visualizations. **(B)** Social reward via NF paradigm (Mathiak et al., 2015). This image has been converted to a gray-scale color scheme from the original. **(C)** An overview picture of our current implementation of the virtual environment as an NF modality. Original environment modified from Epic Games’ project titled “Shooter Game.”

Real-time fMRI enables an online feedback of well-defined network functions of the brain. In a neurofeedback (NF) setting, participants can train to regulate their own brain activity (Sitaram et al., 2017). Thereto, one or several regions of interest (ROIs) are defined, and the participant receives a measure of functional activity from these ROIs with the ex- or implicit task to control the feedback signal, for example, to upregulate or downregulate activation or connectivity (Sulzer et al., 2013). Frequently, target regions are chosen that show dysfunctional activity in populations with psychiatric disorders when compared to healthy individuals (Linden et al., 2012; Orlov et al., 2018; Jaeckle et al., 2019). The voluntary self-regulation can normalize the dysfunctional brain activity and yield symptom reduction (Zweerings et al., 2019). NF using fMRI offers the ability to, theoretically, access deeper brain regions, unlike any other imaging modality (Linden and Turner, 2016). This methodological study focuses on the regulation of the supplementary motor area (SMA). Successful self-regulation of this target has been described before (Scharnowski et al., 2004). One important defining aspect of the efficacy of NF training is the feedback modality. For instance, social reward in the form of virtual avatars smiling at the participants increased the reward value during NF when compared to more traditional modes of NF presentation, such as thermometer representation (Mathiak et al., 2015). The technological flexibility in the recent years allows us to advance NF display modalities and focus on rewarding and motivational qualities of the feedback modality. This offers important advantages particularly in the clinical application of NF paradigms as it yields the potential to improve engagement of participants who suffer from anhedonia or lack motivation. Currently, implemented BCI displays often are rather basic and consist of a number, picture, or temperature bar reflecting the percentage change in the blood oxygen level–dependent signal (BOLD) signal (Bray et al., 2007; Rota et al., 2009; Ruiz et al., 2013) as shown in Figure 1A.

### State of Current Brain–Computer Interfaces

Brain–computer interfaces allow the user to communicate with a device, usually a computer, using their own brain activity or brain connectivity. BCIs are a relatively older technology, and some of their earliest applications are using electroencephalography (EEG) (Lubar and Shouse, 1976). In a medical context, EEG has been used to allow paralyzed and locked-in patients to be able to have rudimentary and simple communication with the outside world (Birbaumer et al., 2006). EEG-based BCIs have been used to treat symptoms of psychiatric disorders such as autism (Jarusiewicz, 2002), obsessive–compulsive disorders (Hammond, 2003), and attention-deficit/hyperactivity disorder (Muñoz et al., 2015). EEG-based BCIs have also been used with VEs to gamify therapeutic methodologies and develop novel treatment methods (Muñoz et al., 2015; Schoneveld et al., 2016). The participants in these settings navigate and perform tasks in a VE and face obstacles in their progress. They must then use mental strategies to solve and overcome these obstacles to move onto the next steps. The drawback of this methodology is that EEG can only target unspecific and large cortical areas. To be able to explore subcortical structures and investigate or target specific and localized activity, we need to use fMRI-based methods.

As more technologically advanced hardware has become available, there have been more VE-based BCIs using rt-fMRI. Such VEs have been used as feedback modalities where participants take a passive role and only view the VE and try to influence aspects of it using their brain activity (Lorenzetti et al., 2018). Lorenzetti et al. (2018) take advantage of the fMRI method and target subcortical areas such as the amygdala. Implementations of NF in VE-BCI using fMRI generally follow a similar methodology (see Figure 1B; Mathiak et al., 2015). Furthermore, even modern fMRI implementations utilize very rudimentary visual feedback modalities (Sorger et al., 2018; Kohl et al., 2019). Hence, we wanted to present an fMRI-based VE-BCI that followed the active role a participant takes, similar to modern EEG implementations (Cohen et al., 2016) while also having retaining the possibility to investigate or target subcortical regions.

### Design Considerations for the VE-BCI

The development and exact nature of a VE depend on the research question and study cohort. One of the most commonly studied effects of video games is on violence and consequently aggressive tendencies (Anderson et al., 2010). Aggression is a serious societal concern and burden, often resulting in harmful consequences by inflicting injury or showing hostility toward other individuals (Anderson and Bushman, 2002). Furthermore, aggression plays a central role as a symptom in many psychiatric disorders (Barratt et al., 1997; Arseneault et al., 2000; Nelson and Trainor, 2007). Accordingly, we chose to design an environment that would support the investigation and modulation of aggressive behaviors.

### Aims and Objectives

The aim of this project was to develop a novel VE for use in the design of rt-fMRI NF-based paradigms. Our first main goal was to create an engaging and immersive environment based on available advanced visuals.

The second major goal was to update the NF presentation modalities and integrate them into the environment. A more meaningful feedback can be achieved this way while the participants navigate and engage with the task in the environment. In a reward-based paradigm, the NF signal, for example, would facilitate participants in accomplishing the task when they are able to regulate their functional brain activity. Conversely, it would hinder participants who are not able to regulate this activity. This platform can train participants implicitly with the NF, without providing them with explicit instructions or education.

The third main goal was automation of the data collection. Usually in experiments using video games as tasks, a considerable amount of time and human resource is invested into labeling and categorizing the task activity and response of the participant (Weber et al., 2006). This platform should cut down these costs by using the VE to log its own content and state in real time with all relevant information.

Following the development of the software platform, an rt-fMRI NF validation study will be run to test the implementation of our goals. Optimization of the experimental design will target the following questions. Are participants able to complete tasks given while actively trying to use NF cognitive strategies? If and how well can participants perceive the NF visualization? How did the environment and NF affect the participant’s mood/engagement? Is there a difference in BOLD regulation in participants performing NF training as compared to participants not performing NF training?

More concisely, the present methodological study should, first, confirm the technical feasibility of a cost-effective VE-BCI setup for NF. Second, we want to test the user interface and assess usability of the setup. Third, we want to confirm the hypothesis that learning of self-regulation was achieved in such a cognitive-demanding setup. The involved neural pattern should be explored, and effects on the participants studied.

## Materials and Methods

### Development and Design of the NF-Based VE

Virtual environments platform development first involved consideration of different programs for VE design. The selection process was narrowed down to freely available gaming engines because they offer flexibility in programming the environments and generally have prebuilt assets that can be used to build a new prototyping environment quickly and effectively. The so-called assets are defined as any single piece of content within the VE, for example, audio files, stimuli, three-dimensional (3D) models, light rays, and animations.

Considering the speed of implementing complex scenarios and the depth of program modifications available, the Unreal Engine 4 (version 4.12.5; Epic Games, Inc., Cary, NC, United States) was selected. Unreal Engine supports one of the most commonly used programming languages “C++,” as well as a visual scripting language called “Blueprints,” both of which offer a high level of functional control over the environment. Both these languages offer certain exclusive advantages. C++ gives a much more flexible control over the logic of the VE, whereas Blueprints scripting offers a more intuitive interface that can be used to quickly prototype an environment. Visual aspects such as animations and user interfaces are simpler to program in Blueprints, whereas C++ makes implementing environment logic and behavior more accessible. Unreal Engine 4 offers protocols to communicate between the two programming languages, adding more flexibility in designing VEs. Furthermore, Unreal Engine 4 has a very active and diverse asset store, which offers many prebuilt assets, which can significantly decrease the amount of time required to program a task or VE. Many of these assets are free and open source. While available primarily as learning resources, Epic Games encourages their use as templates to build upon. Additionally, for future extensions, the engine offers plug-ins that allow the environments to be transferred into virtual reality (VR) and can be used with VR headsets.

As one of the goals of the environment was to facilitate tasks involving violence and aggression, we chose to create a first-person shooter (FPS) for the project. Epic Games offers a complete game titled “Shooter Game” as a learning experience and example to demonstrate the capabilities and resources of the Unreal gaming engine. We used the Shooter Game as a template to build our environment.

The Shooter Game is designed as an arena shooter. In this game, as is typical in arena shooters, the players are divided into two groups that will fight each other to earn the most points before a timer runs out. Points are generally earned by successfully eliminating enemy team players and by successfully avoiding elimination from the enemy team. We did not modify the point earning aspect of the game; however, the game difficulty was tuned to make it easier for players to navigate and play the game. This was done to offset the increased difficulty from the restrictive space inside an MRI scanner, where a full range of motion with regular input devices is not possible. Having chosen a visually pleasing and up-to-date environment, the NF modalities were developed and integrated in this environment (see Figure 1C).

All software and similar resources used in the experiment are available on request from the authors.

### Closed-Loop Feedback Modalities

In the present study, we want to apply a closed-loop feedback of brain activity within the VE that can act implicitly (Watanabe et al., 2017). Feedback by the presentation of numbers or simple bars/thermometer displays requires explicit evaluation of the stimuli (Rota et al., 2009). In contrast, a VE enables implicit reinforcement (Richardson and Waller, 2007) of self-regulation by making successful game events more likely. For instance, better visibility increases the likelihood of hitting a target and can be achieved with relative ease by increasing an otherwise dim lighting. We investigated different possibilities to shape the FB modality as described in the following.

#### Visibility/Luminosity

**FIGURE 2:**
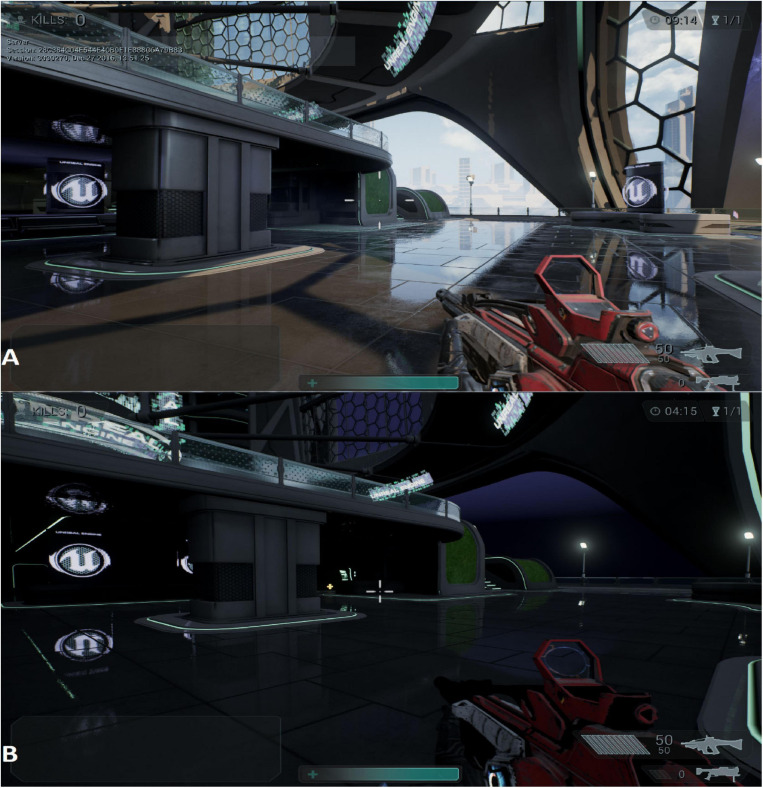
Modified version of the “Shooter Game” using visibility as the NF modality. **(A)** The maximum luminosity condition and running crosshair bars are illustrated, whereas in **(B)** the luminosity is set to minimum, and the crosshair bars while the player character is stationary are illustrated.

The first approach to design the NF modality was the manipulation of the lighting in the environment as a function of the BOLD signal from the ROI. Accordingly, a system was implemented that could modify luminosity of the environment in real time. All the existing lighting and reflection captures were removed or modified in order to achieve the desired control over luminosity. The global lighting settings were adapted to create a more convincing dark environment, and a new dynamic directional light was introduced as a simulated sun light. This light source was programmed to be modulated online by the real-time fMRI processing toolbox. Thereby, the neural signal can be fed back to the participant to yield a NF setup. The implemented setup is shown in Figure 2. The logic of the environment is programmed to decrease the luminosity (and hence the visibility) when the NF BOLD signal is lower than the baseline value, or conversely increase when the NF BOLD signal is higher than the baseline value of the targeted ROI. A signal inversion is also possible by changing one variable in the implemented program. Hence, the participant has to either maintain or hyperactivate/hypoactivate the target ROI to perceive the environment easily and facilitate in completion of their VE task. The lowest possible visibility condition does not prevent the participant from completing the task but increases the difficulty.

An additional modification was made in this modality. In aggression-related paradigms, where a computer-controlled “bot” enemy would be used in this environment, the artificial intelligence (AI) of this bot needed to be modified. In the original Shooter Game arena, the bot immediately engages the player character upon vision of the player. This means that no matter how far the bot is from the player character, it will fire upon the player if it has line of sight. However, in the lower visibility environment, this can actually be an advantage to the player, as it becomes easier to spot a weapon being fired in a dark arena. To counter this, the bot’s AI is programmed to change behavior after a certain level of luminosity is reached. The bot in this case tries to get closer to the participant before engaging in combat. This modification retains the difficulty to navigate the environment in low luminosity and especially in spotting a faraway bot, which is much easier to spot at high luminosity. However, if the bot is spotted during low luminosity at a faraway distance and shot upon, it will start to retaliate as usual. It is also possible in the VE to not change this behavior of the AI even in low luminosity.

#### Accuracy

The second approach for the NF modality was manipulating the shooting accuracy of the player character during the VE task. There is a specific shooting mechanism for the weapons in the Shooter Game software. Every time a character shoots, he/she does not shoot in a straight line at the center of the crosshair; rather, there is a small invisible circle drawn around the center of the crosshair, and the bullet shoots at a random place within this circle. Therefore, the bigger the size of the circle, the less accurate the weapon becomes. The size of this circle is communicated to the player via the four crosshair bars surrounding the central dot, as shown in Figure 2. This is a measure of the size of the circle for the player. The bars move either closer to or farther away from the dot, depending on the current size of the circle, as shown in Figure 2. Activities such as running and not using the weapon sight increase the size of the circle. Conversely, standing still and aiming using the sight decrease the size of the circle and hence increase the accuracy.

The size of the aiming circle was modified based on the NF BOLD signal in this condition. The aiming circle was programmed to decrease in size when the NF BOLD signal was higher than the baseline value and to increase in size when the NF BOLD signal value was lower than the baseline. Signal inversion is possible with a change of one variable in the program or implementation of other transfer functions.

#### Movement Speed

The third approach for the NF modality was to manipulate the movement speed of the player character within the VE. Thereby, the speed that the player’s VE character can achieve depends on the regulation signal. The movement activity remains under the control of the player with a keyboard, but when the NF BOLD signal is lower than the baseline, the player can only move at slower speeds, whereas during phases with high feedback signal, the player can outmatch the initial speed assigned to the player character. A signal inversion is also possible in this modality.

In competitive paradigms, increased speed supports the participants in accomplishing their tasks faster, avoiding hostiles with more ease, and getting into advantageous positions quicker and easier. Decreased speed, on the other hand, greatly increases the chance of getting shot and not being able to get into advantageous positions before being engaged.

### Real-Time Data Processing and Logging

For online image data processing and extraction of the feedback signal, we utilized a MATLAB (Release R2016a, version 9.0; MathWorks, Inc., Natick, MA, United States) toolbox that was tested in previous fMRI NF studies in our laboratory (Koush et al., 2012). The EPI sequence used for the NF measurements had a repetition time (TR) of 1 s. The SMA was defined anatomically for each participant, and an individualized mask created. Signal intensity averaged over the whole SMA mask was extracted via the toolbox. An exponential moving average filter was used to detrend the data, as well as a Kalman filter for spike removal. Both filters used two time points, current and previous volume (*t* and *t* – 1), to filter the signal between approximately 0.003 and 0.1 Hz. This filtered signal was then normalized for the VE; this is elucidated in detail under the NF signal section. All of the steps were performed within 1 TR after the latest volume was received. Hence, the NF signal in the VE was updated once every TR. The details of BOLD signal extraction and calculation are described in a previous publication (Koush et al., 2012).

These MATLAB routines are originally based on the SPM software (Statistical Parametric Mapping 2012, SPM12^1^) and were further complemented with routines for real-time communication with the game. The routines sent the processed NF signal using transmission control protocol over a local host to the VE. There, it was time-stamped, protocoled, and displayed as feedback using the modifications to the VE parameters.

A real-time logging function was programmed into the VE. This function keeps track of predefined events within the game. All events are timestamped with an accuracy of 8 ms. There are some unspecific events that the VE is programmed to always track. Other than these events, it is also possible to record paradigm-specific actions. These need to be programmed into the environment before it is used experimentally. All tracked actions are saved as and organized into separate text files with a “.sav” extension. These text files are comma delimited for ease of import into various data analysis software as shown in Table 1. Each recorded event is always followed by a timestamp at the end. The timestamp records the time since the start of the VE session.

**TABLE 1:** Predefined events logged by the VE.

Number	Event	Description	Example (readable) text log
1	Location of the player character	Cartesian coordinates from the center of the map [m], with timestamp [s]	*X*, 5480.95 m, *Y*, 4935.93 m, *Z*, 848.14 m, 137.29 s
2	Facing direction of the player character	Rotation [degrees] of the player character camera, with timestamp [s]	Yaw, 53.51°, Pitch, 355.84°, 25.62 s
3	Location of the bot	Cartesian coordinates from the center of the map [m], with timestamp [s]	*X*, 1466.81 m, *Y*, −980.90 m, *Z*, 212.15 m, 136.79 s
4	Key presses of the player	With “key pressed” and “key released” times [s]	Strt_LM, 69.58 s Stop_F, 73.02 s Stop_L, 132.81 s
5	NF BOLD signal data	Real-time processed BOLD signal with timestamp [s]	0.71268, 47.66 s
6	Game event: player character shoots	Each bullet shot by the player character with timestamp [s]	P_Bullets, 3, 51.70 s
7	Game event: bot shoots	Each bullet shot by the bot with timestamp [s]	AI_Bullets, 5, 32.14 s
8	Game event: player character being hit	Player character being hit by a bot bullet with timestamp [s]	PlayerHit, 2, 32.14 s
9	Game event: bot being hit	Bot being hit by a player character bullet with timestamp [s]	EnemyHit, 2, 51.70 s
10	Game event: player character death	Player character dying due to running out of health with timestamp [s]	Death, 2, 510.34 s
11	Game event: bot death	Bot dying due to running out of health with timestamp [s]	Kill, 1, 52.11 s
12	Game event: health picked up	Player character picks up the health package to replenish health completely with timestamp [s]	H_pickup, 9, 614.17 s
13	MRI trigger signal	To sync the MR scanned volumes with the VE, with timestamp [s]	MRT, 33.46 s

### Participants

In total, 24 participants took part in the experiment (five females; age 24.0 ± 2.8 years). Participants were recruited using flyers placed on notice boards at the RWTH University Hospital Aachen. All volunteering participants were screened for inclusion and exclusion criteria before being admitted to the study. Inclusion criteria were experience with video games (min ⋅ 1 h/week of play and ≥5 years of gaming) inclusive of experience with shooting games. Video game players were chosen because they are used to navigating VEs outside the MRI setting and would have reduced difficulty in familiarizing themselves with the VE platform and the input devices.

Exclusion criteria were acute psychiatric, neurological, and medical disorders, as well as contraindications to MRI. All participants had normal or corrected to normal vision, no contraindications against magnetic resonance investigation, and no neurological or psychiatric illness as assessed by the German Structured Clinical Interview for the *Diagnostic and Statistical Manual of Mental Disorders, Fourth Edition* screening questionnaire (Wittchen et al., 1997). All participants were right-handed according to the Edinburgh Handedness Inventory (Oldfield, 1971). The study protocol was approved by the Independent Ethics Committee of the University Hospital RWTH Aachen (EK 188/17), and written informed consent was obtained from all participants after description of the study to them.

### Experimental Procedure

We conducted a randomized, single-blind study. The participants were assigned into one of two groups based on a randomization list and were blind to their group allocation: the active NF group (*n* = 12) and the reduced contingency (rc-)NF group (*n* = 12; Table 2). The study took place on 2 separate days. After recruiting the participants by telecommunication, we scheduled a training day and a measurement day. On the training day, participants were screened for inclusion and exclusion criteria and informed in verbal and written form about the purpose of the study and signed the consent form. Further, demographic data were collected. Subsequently, the participants were trained on the VE and on the hardware input devices outside the MRI scanner. This was done to familiarize the participants with the new VE and to train them how to control the input devices.

**TABLE 2:** Group demographics.

	NF	rc-NF	Statistics
*n* (male/female)	12 (10/2)	12 (9/3)	χ^2^ (1, *n* = 24) = 0.25, *p* = 0.62
Age	26.4 ± 5.4*years*	24.8 ± 3.6^y^	*t*(22) = −0.85, *p* = 0.41
Education	15.4 ± 2.7^y^	15.3 ± 3.0^y^	*t*(22) = −0.14, *p* = 0.89
Gaming since	17.8 ± 6.2^y^	15.5 ± 4.9^y^	*t*(22) = −1.03, *p* = 0.32

The second day (measurement day) was scheduled latest 3 days after the training day to ensure that the participants could still recall what they had learned on the training day. The total measurement time inside the scanner lasted about 90 min. During the measurement, each participant took part in three VE NF runs. Each run was 10 min long. Each VE run was preceded and followed by a Self-Assessment Manikin (SAM) valence and arousal rating (Bradley and Lang, 1994).

**FIGURE 3:**
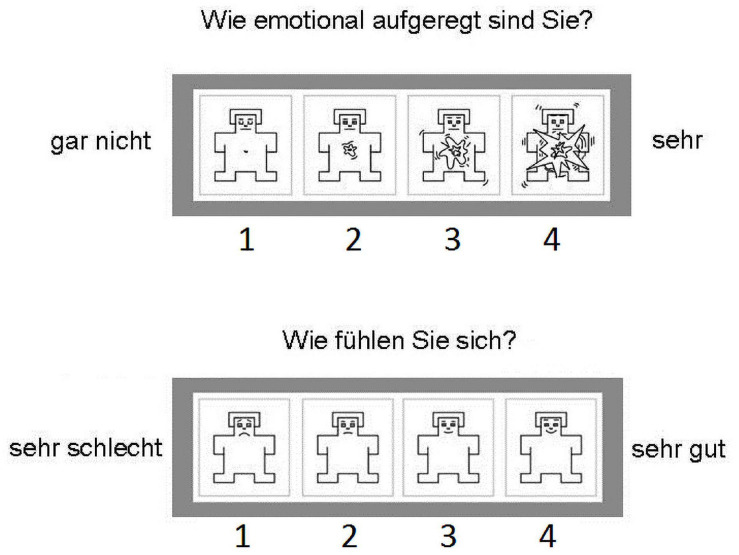
The modified SAM rating scales as administered to the participants in the scanner. The participants could select directly one of the options on the four input buttons. The *top scale* is the arousal rating. The question above it asks, “How emotionally excited are you?” The options range from *left* “not at all” to *right* “very.” The *bottom scale* is the valence rating. The question above it asks, “How do you feel?” The options range from *left* “very bad” to *right* “very good.”

**FIGURE 4:**
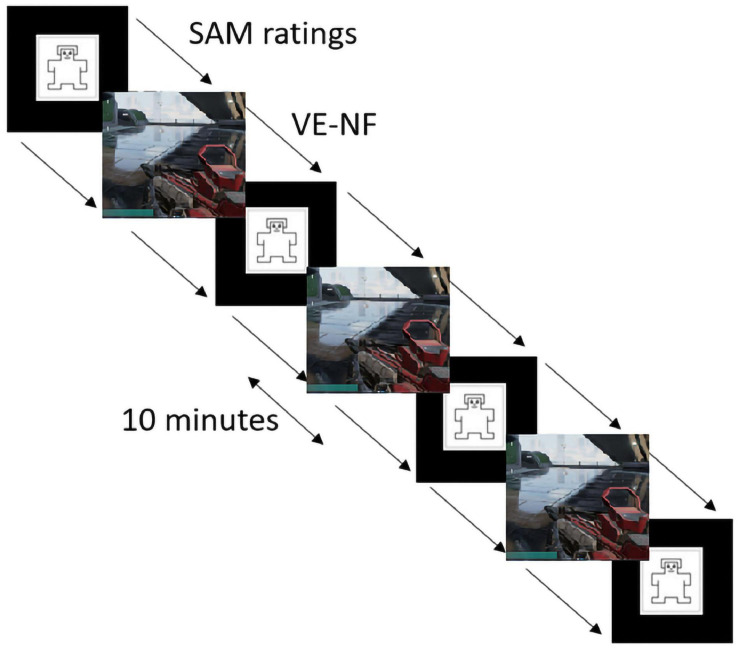
Experimental design of the gaming sessions and SAM ratings (Bradley and Lang, 1994) within the scanner.

The SAM ratings were modified to a 4-point Likert scale from a 9-point Likert scale. This reduction facilitated the responses on a four-button response box within the MRI scanner. The modified scales used are illustrated in Figure 3. The SAM ratings were displayed to the participants via the Presentation software (Neurobehavioral Systems, Inc., Berkeley, CA, United States). Figure 4 illustrates the experimental design.

All participants received the same standardized instructions. They were instructed to achieve the highest possible score during the VE runs. It was made clear that achieving the highest score meant having the highest number of kills and the lowest number of deaths during each run. The participants were also instructed to regulate their brain activity, and they were informed that if they were successful in doing so, their character in the VE would become faster. Each participant was free to choose whichever NF cognitive strategy would work best for them. However, they were also given three strategies as guidelines to help them prepare for NF training. These three strategies were as follows: (1) imagining body movement, (2) relaxing and peaceful thoughts, or (3) thinking about spoken speech. The participants were free to use these strategies and evaluate their effectiveness or use their own. After each NF run, the participants were interviewed and asked which strategies they used. After the complete measurement, the participants filled out self-reported measures and were debriefed in a following interview.

### Materials and Data Acquisition

An MR-compatible and custom-modified Microsoft D67 optical trackball was used during the MRI scanning as the mouse input. An MR-compatible LUMItouch (Photon Control Inc., Burnaby, BC, Canada) four-button response box was used as the keyboard input device in the MRI scanner. An MRI-compatible monitor screen (BOLDscreen; Cambridge Research Systems Ltd., Rochester, United Kingdom) displayed the VE to the participants. The screen was 24 inches (60.96 cm) diagonally, displayed the VE on a native 1,900 × 1,200 pixel resolution, and was viewed by the participant using a mirror. The participants’ eyes were approximately 14 cm from the mirror, and the distance between the mirror and the screen was 144 cm. The participants heard audio using the OptoACTIVE II^TM^ MR-compatible headphones (Optoacoustics Ltd., Mazor, Israel).

A 3-T Siemens-Prisma fit scanner was used for the measurements. Each gaming session was acquired using a multiband multiecho echo planar imaging sequence with the following parameters: TR = 1,000 ms; echo time = 13, 28, 43, and 57 ms; matrix = 64 × 64; iPAT factor for parallel imaging = 2 (GRAPPA); slices = 36 with threefold multiband acceleration; 610 repetitions; flip angle = 67°; and transversal slice orientation.

### Measures and Statistical Analysis

To address our validation design questions, we investigated measures for task performance, subjective experience of neural self-regulation, allover acceptance of the paradigm, and objective measure of changes in the NF paradigm.

#### Task Performance

For assessing task performance, two measures were applied: first, the relation between player kills and player deaths and, second, player shooting accuracy. In this type of first-person arena shooters, the relation between kills and deaths is a common metric of performance and is often used to determine how individuals and teams perform during professional and amateur FPS gaming matches (Shim et al., 2011). For this analysis, the same kill-to-death relation metric was used as is preprogrammed into the Shooter Game. This relation is calculated as a score by multiplying the amount of kills the player has achieved by two and then subtracting the amount of deaths that the player has accrued (equation A). This is a good representation of performance as it shows skill at focusing in situations demanding quick reactions and avoiding danger, as well as navigating the environment to find advantageous spots for improving the player’s score. Accuracy was calculated by dividing the number of bullets the player hit onto the bot by the total number of bullets shot by the player and then converting it into a percentage (equation B).

S⁢c⁢o⁢r⁢e=(P⁢l⁢a⁢y⁢e⁢r⁢k⁢i⁢l⁢l⁢s×2)-P⁢l⁢a⁢y⁢e⁢r⁢d⁢e⁢a⁢t⁢h⁢s     (A)

$$A\,c\,c\,u\,r\,a\,c\,y=100\times \left(\frac{S\,h\,o\,t\,s\,h\,i\,t\,b\,y\,p\,l\,a\,y\,e\,r}{A\,l\,l\,s\,h\,o\,t\,s\,b\,y\,p\,l\,a\,y\,e\,r}\right)\;\left(B\right)$$

A brief interview conducted after each VE-NF session assessed the use of NF strategies the participants applied during the session. The interview consisted of two questions: (1) “Which strategies did you employ to influence your brain activity?” and “How successful was the application of the strategy/strategies?” Keywords from the responses were noted.

#### Perception on Contingency of Neural Regulation

At the end of the fMRI measurement day, the ease of perception of the NF modality was assessed. Participants rated their subjective assessment of control over their regulation, and hence over the NF modality, on a 10-point Likert scale.

#### Emotion Effects and Acceptance

The Positive and Negative Affect Schedule (PANAS) was applied before and after the measurement to determine any affect from the environment and NF training on the mood of the player. Furthermore, the SAM assessed arousal and valence before and after each VE-NF session.

#### NF Signal

The ROI chosen for NF was the SMA as provided by the Automated Anatomical Labeling (AAL) atlas within the Wake Forest University Pickatlas toolbox^2^ (version 3.0.5b). The SMA is a very well-studied brain region and has been shown to be responsible for planning (Pfurtscheller and Berghold, 1989) and initiation of voluntary motion (Kornhuber and Deecke, 1965). Furthermore, the SMA has also been established as an ROI in previous NF work where motor imagery was used to regulate the functional activity (Hwang et al., 2009; Hampson et al., 2011; Buyukturkoglu et al., 2013). Hence, the SMA was chosen, as it is a region already established in NF paradigms and one that is shown to be reliably controllable. The aim was to reduce the difficulty in regulation of brain function such that it would not become a bottleneck in assessing the VE-based NF.

For this study, we chose to use movement speed as the NF modality. In a behavioral prestudy, the effects of the three NF modalities were tested. The movement speed modality resulted in the most effective feedback as it led to the most disadvantageous change in the VE when the control signal was lowered. Reduced movement speed hindered the ability to dodge the enemy attacks as well as to relocate toward safe or advantageous positions most significantly.

The control range for the NF signal ranged from slow movement speed (about 40% of the maximum speed) to the fastest movement speed in the VE. The latter was also the maximum speed of the AI controlled enemy bot. For contrasting specific NF effects, the participants were assigned either to the active NF or to the rc-NF group. In the active NF group, the participants received regular feedback; i.e., a 1% change in BOLD signal activity was scaled to a 100% change in the NF signal. In the rc-NF group, the participants received an imperceptible feedback, i.e., a 10% change in BOLD signal activity was scaled to 100% change in the NF signal. The percentage change in the BOLD signal was calculated against a baseline measure during the start of a VE-NF run where the participants could view and move around the VE but not interact with it.

Because of the interactive nature of the gameplay, no set time courses of gaming behaviors or BOLD responses can be given. Therefore, we discarded the phase information of the signal and calculated the frequency spectra of the BOLD signals in the feedback ROI in each participant. The individual SMA time courses were extracted for each participant. The time courses were then averaged within their respective groups, resulting in one averaged time course for the NF group and one for the rc-NF group. A fast Fourier transform (FFT) was performed over each average time course. The power was then calculated as the absolute magnitude squared of the FFT. Differences in the power distribution between feedback and rc-NF groups were analyzed.

#### Connectivity Analysis

To test the effects of the localized SMA regulation on task networks, in particular, the hippocampus involved in the virtual navigation task, a connectivity analysis was performed (MATLAB toolbox CONN release 19b; Whitfield-Gabrieli and Nieto-Castanon, 2012). To minimize T1 saturation effects, the first 15 volumes of each task session were removed from the analysis. All preprocessing of our fMRI data was also performed in CONN. The preprocessing steps included 3D motion correction, Gaussian spatial smoothing (12-mm full-width half-maximum kernel), linear trend removal, and bandpass filtering. The functional images were coregistered to the anatomical volume. The image data were normalized to Montreal Neurological Institute (MNI) space. The standard pipeline for confound removal in CONN was performed next: The rigid body transformations of the six motion parameters and their first-order derivatives, the global signal time courses, and the individual time courses of white matter and cerebrospinal fluid were extracted and regressed out. Subsequently, seed-based connectivity analysis was performed. Reflecting the NF learning, the real-time SMA served as a seed region. ROI-to-ROI analysis was performed between the SMA and the bilateral hippocampus. Connectivity of the SMA regulation target with the left and right hippocampi (anatomical definition from the AAL atlas) was compared between NF and rc-NF conditions. The cluster-to-cluster analysis applied Bonferroni corrected thresholds. The seed-to-voxel mapping applied an exploratory-voxel threshold according to *p* < 0.001.

#### Motion Parameters

Head movement may be a very natural aspect of engaging in FPSs in a regular setting. This was hence one of the concerns using such a VE in an fMRI setting. Although the participants were instructed to not move their heads in reaction to the game, complete compliance cannot be assumed. Therefore, we extracted and analyzed the motion parameters from the functional connectivity toolbox CONN.

#### Statistical Analysis

The remaining analyses were performed with the open-source statistics software JASP (JASP Team, 2019, Windows version 0.11.1, University of Amsterdam, the Netherlands), and using MATLAB (release R2018a, version 9.4.0; MathWorks, Inc., Natick, MA, United States). For task performance, the differences between groups (i.e., group effect) and within subjects (i.e., session effect) were analyzed using a repeated-measures analysis of variance (ANOVA) with factor “in-game performance.” In case the ANOVA revealed significance, *post hoc* paired-samples *t*-tests were performed to investigate the session effect. An independent-samples *t* test was performed to investigate the perceived control on neural regulation. One-way repeated-measures ANOVAs were conducted to assess group effect and session effect on PANAS scores and SAM ratings. An independent-samples *t*-test was performed on the frequency spectra of the NF and rc-NF group averaged BOLD time courses to explore differences in neural regulation. A *p* < 0.05 was considered significant.

## Results

### Task Performance

**FIGURE 5:**
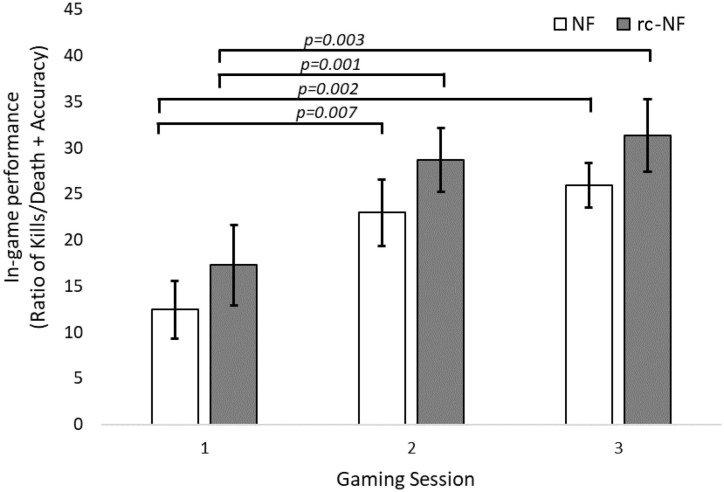
Gaming performance over three gaming sessions shows a learning curve (Ritter and Schooler, 2001). Participants improve over time while still performing NF-based regulation via cognitive strategies.

The primary VE task of the participants was to maximize their in-game score, i.e., to eliminate the AI controlled enemy bot as much as possible and in parallel avoid being eliminated. A repeated-measures ANOVA revealed that the participants were not only able to complete the VE tasks but also significantly improve [*F*(2,44) = 23.56, *p* < 0.001] their performance metric over the three VE NF sessions. There was no significant difference between the two groups [*F*(1,22) = 1.48, *p* = 0.24] or an interaction [*F*(2,44) = 0.02, *p* > 0.9]. The paired-samples *t* tests revealed that the NF and the rc-NF group improved significantly [NF: *t*(11) = 3.99, *p* = 0.002, *d* = 1.151; rc-NF: *t*(11) = 3.72, *p* = 0.003, *d* = 1.074] from the end of the first session to the end of the final VE session as shown in Figure 5.

**FIGURE 6:**
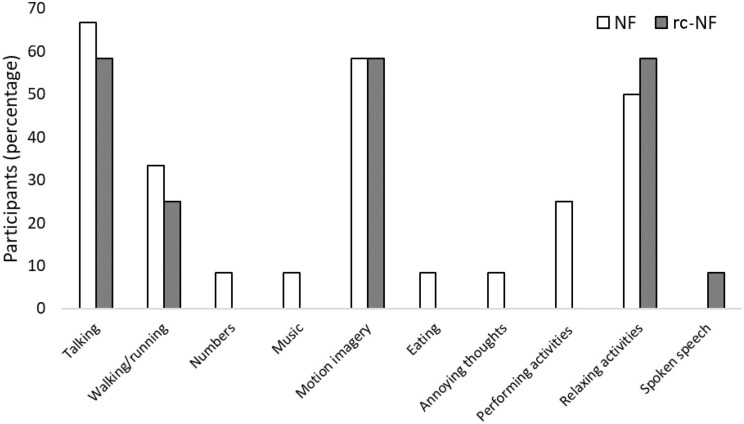
Imagery strategies for NF employed by the participants.

After the measurement, participants were asked about their use of NF cognitive strategies. The most commonly used NF strategy was imagining talking or imagining a discussion (NF: 67%, rc-NF: 58%). This was followed by imagining general movement (NF: 58%, rc-NF: 58%) and imagining relaxing activities or situations (NF: 50%, rc-NF: 58%) as shown in Figure 6. Participants in the NF group also employed more and varied NF strategies, as they attempted 9 of the 10 reported strategies. Comparatively, the rc-NF group attempted only five strategies. All individuals (*N* = 24) reported successfully employing NF strategies. However, 33.3% of participants reported that they believed that the strategies had no perceivable effect on their movement speed and/or task performance.

### Perception on Contingency of Neural Regulation

**FIGURE 7:**
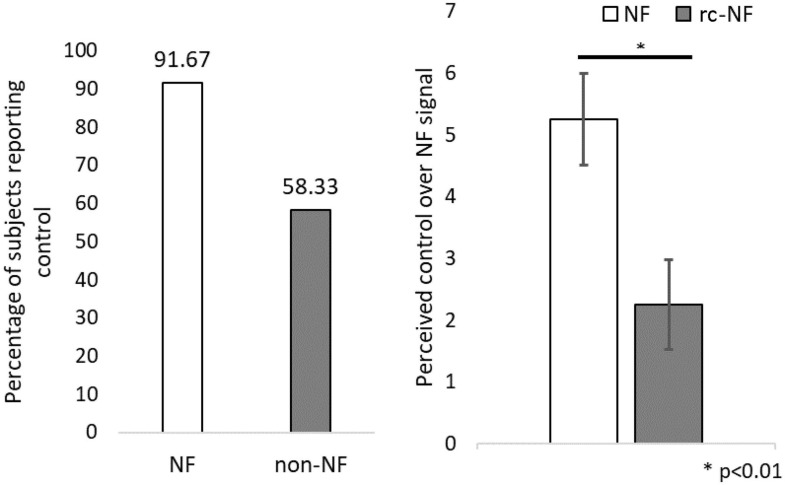
**(Left)** Participants reporting if they perceived control over the NF modality. **(Right)** Amount of perceived control over neural regulation. ^∗^*p* < 0.01.

Concerning the subjective ability to control the NF signal (Figure 7), 91.7% of participants in the NF group reported to have control over the feedback signal, whereas only 58.3% of participants in the rc-NF group reported the same. Further investigation revealed that of the 91.7% of participants in the NF group, most participants (63.6%) rated the perceived control over their NF regulation as high. In comparison, of the 63.6% of participants in the rc-NF group, only 28.6% rated a high control amount. Perceived control amount was categorized as high if it was rated higher than 5 on the 10-point Likert scale. The NF group (mean = 5.25, *SD* = 2.56) compared to the rc-NF group (mean = 2.25, *SD* = 2.53) perceived a significantly higher control over their neural regulation [*t*(22) = −2.89, *p* = 0.009, *d* = −1.179], as depicted in Figure 7.

### Emotion Effects and Acceptance

**FIGURE 8:**
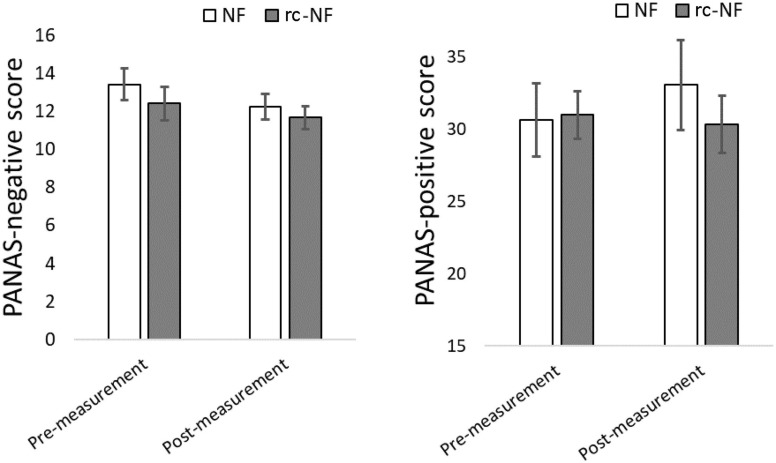
**(Left)** PANAS negative affect decreased on a trend level for both groups after the NF-training. **(Right)** A trend to a less negative and a more positive affect change in the NF group emerged, but the comparisons failed significance.

Repeated-measures ANOVA revealed that there was no main effect of the VE-NF training on the PANAS [PA: *F*(1,22) = 0.48, *p* = 0.49; NA: *F*(1,22) = 2.21, *p* = 0.15] or an interaction [PA: *F*(1,22) = 1.50, *p* = 0.23; NA: *F*(1,22) = 0.10, *p* = 0.75]. However, considering the data, both groups decrease in the NA after the measurement versus before the measurement. For positive affect, the NF group shows an increase in the PA, whereas the rc-NF group shows a decrease (Figure 8).

**FIGURE 9:**
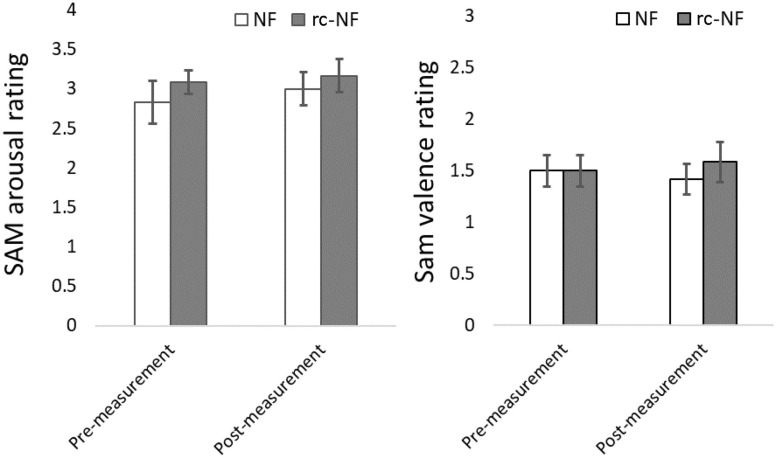
**(Left)** SAM ratings showing a trend toward more arousal after the VE-NF in both groups but failing significance. **(Right)** SAM valence ratings did not reveal a difference between premeasurement and postmeasurement. Note that the SAM was simplified to a 4-point Likert scale.

Repeated-measures ANOVA of SAM arousal ratings revealed an increase in the arousal of the participants [*F*(1,22) = 0.37, *p* = 0.55] as the main effect of the VE-NF training but failed significance (Figure 9). There were no significant differences between the two groups [*F*(1,22) = 0.88, *p* = 0.36] or a significant interaction [*F*(1,22) = 0.04, *p* = 0.84].

A repeated-measures ANOVA of the SAM valence ratings revealed no effect of the VE-NF training [*F*(1,22) = 0.00, *p* = 1.00]. There was no significant difference between the two groups [*F*(1,22) = 0.185, *p* = 0.67] or a significant interaction [*F*(1,22) = 0.47, *p* = 0.50].

### NF Signal

**FIGURE 10:**
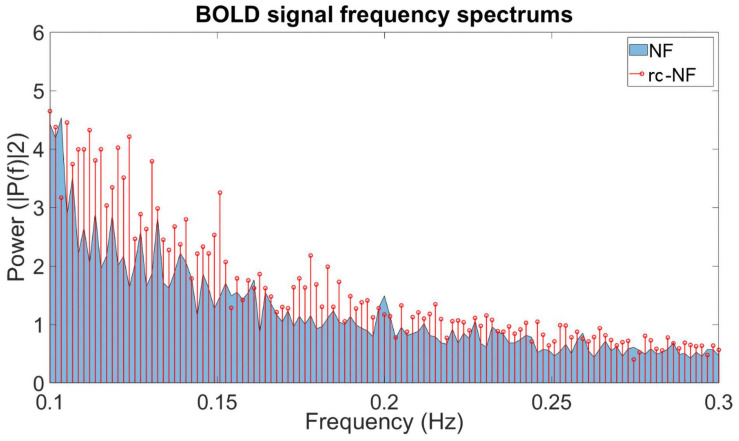
Frequency plot showing the contribution of different frequencies in the BOLD signal from the ROI during the NF training. The NF group tends to emphasize different frequency ranges during regulation compared to the rc-NF group.

**FIGURE 11:**
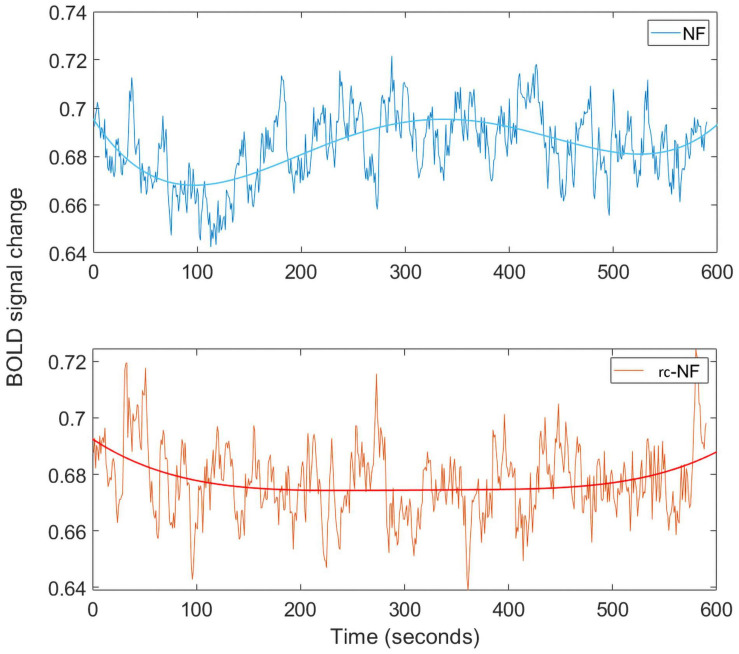
**(Top)** Averaged BOLD signal for the NF condition throughout the time course. The BOLD signal from our ROI has been processed using the same pipeline as in the rt-fMRI toolbox. **(Bottom)** Averaged BOLD signal for the rc-NF condition throughout the time course. Fitting applied a fourth-degree polynomial.

We investigated the specific influence of the NF condition on the BOLD signal in the target ROI (Figure 10). The frequency spectra of both groups differed significantly in a high-frequency band [0.1–0.2 Hz: *t*(70) = −2.2463, *p* = 0.0278; Figure 10]. In this methodological development study, no association with specific events was investigated, but preliminary evidence suggests that the NF group employed different regulation strategies compared to the rc-NF group. This is illustrated in the averaged response curves of both groups (Figure 11). Importantly, the quantitative differences in the time courses confirmed the contingency of the NF signal in the current setup.

### Functional Connectivity

**FIGURE 12:**
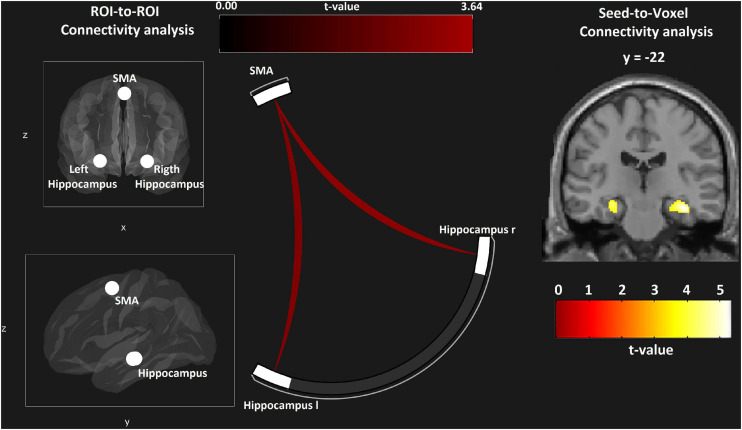
ROI-to-ROI analysis reveals connectivity differences between the two learning groups with both hippocampal regions **(left and center panel)**. In the seed-to-voxel analysis, selective effects at the hippocampus are suggested, matching the involvement with the VE navigation task (voxel-wise threshold *p* < 0.001; **right panel**).

The hippocampus is involved in path planning and mental navigation as required by the VE task. We performed an ROI-to-ROI analysis to investigate the functional connectivity between the SMA and the bilateral hippocampus. In the group comparison rc-NF > NF, functional connectivity was significantly higher in the rc-NF compared to the NF group [SMA–left hippocampus: *t*(22) = 3.56, *p* = 0.0035; SMA–right hippocampus: *t*(22) = 3.64, *p* = 0.0029; Figure 12]. Further, we explored connectivity differences between the two NF groups in a seed-to-voxel analysis. At a voxel-wise threshold according to *p* < 0.001, one cluster each emerged in the left hippocampus (MNI: *x* = −26, *y* = −22, *z* = −16; *T* = 4.27, *k* = 35, *p*_*FWE*_ = 0.02) and the right hippocampus (MNI: *x* = + 36, *y* = −22, *z* = −18; *T* = 5.19, *k* = 149, *p*_*FWE*_ = 0.004; see insert in Figure 12). Small-volume correction with the hippocampus (*a priori* AAL atlas) confirmed the location of these clusters in the hippocampus with a *p*_*FWE*_ < 0.05.

### Motion Parameters

**FIGURE 13:**
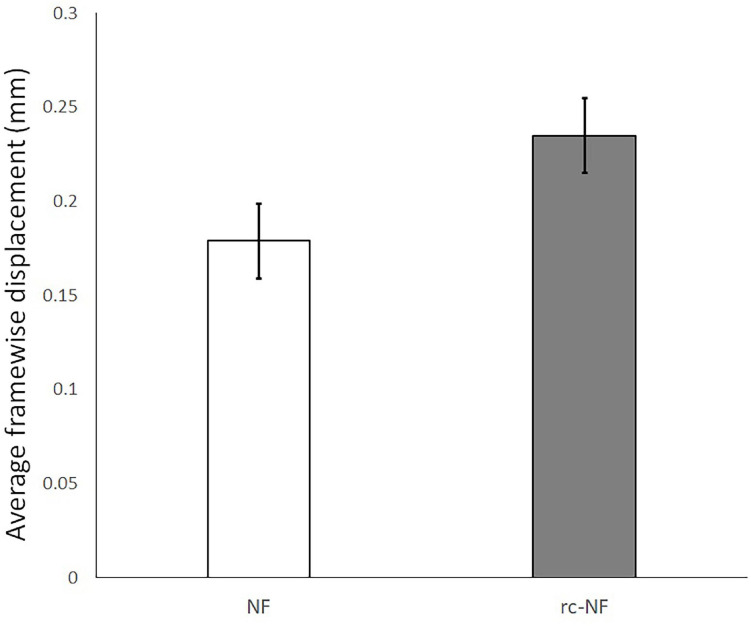
Average framewise displacement with 95% confidence interval in the NF and the rc-NF groups. Head motion seems to be rather lower than in previous fMRI studies on VEs or even non-motor tasks (Mathiak and Weber, 2006; Osterbauer et al., 2006).

We calculated the average framewise displacement of the participants and compared the groups. The average displacement for the NF group was 0.18 ± 0.07 mm and for the rc-NF group was 0.24 ± 0.07 mm (Figure 13). While the two groups were not significantly different [*t*(22) = 2.01, *p* = 0.056; *d* = 0.822], there was a trend to less motion in the effective NF group, and both were below 0.3-mm average framewise displacement and smaller than previously reported values (Osterbauer et al., 2006). Considering the maximum framewise displacement, only 2 of the 24 participants had a displacement higher than 3 mm.

## Discussion

We present a VE for closed-loop NF based on fMRI. The VE can simulate social actions and interactions and is modulated by feedback from anatomically defined regions. The modulation led to contingent changes in reward probability inducing behavioral changes. In a single-blind randomized pilot study, closed-loop NF demonstrated a significant effect on the perceived level of control and on neural patterns in the SMA when comparing a high- versus low-gain condition. We confirmed for the first time the feasibility and effectiveness of such VE-based regional NF during rt-fMRI. An engaging and immersive VE was created, taking into consideration all the modern technologies available currently. The immersion led to participants being motivated to complete the task within the VE. Implicit NF stimuli were implemented in the VE, creating a novel and meaningful NF signal, which facilitated the participants to use NF cognitive strategies. This also increased participant motivation for NF as successful regulation of the BOLD signal resulted in easing the VE tasks. Furthermore, the VE was programmed to log all necessary data in real time, reducing time investment and personnel overhead. The successful implementation of the BCI allows us to design future studies where we can study virtual aggression in a modern VE.

### VEs: An Important Tool in Neuroscientific Experiments

Virtual and gaming environments have been used vigorously in neuroscientific experiments. Specifically, they are used as a means to investigate, test, or hypothesize mechanisms underlying psychological behaviors and disorders. The Penn Computerized Neurocognitive Battery is an acclaimed test battery that assesses performance of cognitive domains such as social cognition, complex cognition, and sensorimotor speed (Moore et al., 2015). There are several tests in the battery that implement video game design in rudimentary visual modalities. For example, in the motor praxis test, the participant interacts with a graphical user interface and is given a limited amount of time to quickly navigate and click on as many boxes that appear in a random place within the playing area. The Cyberball test is another example of rudimentary visualization in video game form of a game of catch. The participant is shown his own character present with two other player characters. The test mainly assesses social stress by including or excluding the participant from the game using the other two computer-controlled “player” characters (Wagels et al., 2017). The Balloon analog risk task is one of the simplest visualizations of a gamified task, where a participant must evaluate the risk between securing the reward he has earned or going further, earning a higher reward with a higher chance of losing all of the reward (Lejuez et al., 2002). This task mainly assess risk taking and has even been used to study the influence of genetic variance in brain function for risk taking (Rao et al., 2018).

Advanced implementations of VEs have been further used to validate and test efficacy of various therapeutic interventions in clinical populations. Avatars within VEs have been used to embody participants and create agency to help promote weight loss strategies in overweight populations (Johnston et al., 2012; Behm-Morawitz et al., 2016). Alcohol-dependent patients have undergone VE training to resist social pressure and craving cues and develop coping skills (Lee et al., 2008). Hence, VEs are a vital tool for studying various disorders, their underlying mechanisms, and testing therapeutic intervention feasibility.

### Contingency of the Feedback Signal and Task Performance

The NF stimulus modality was successfully perceived by the participants, and they were subjectively successful in regulating their brain activity without significant impediment of gaming performance. The cognitive NF strategies interview indicated employment of imagery strategies from all the participants and a subjective feeling of control over the feedback modality. Failure of self-regulation is associated with excess of subcortical or emotional involvement (Heatherton and Wagner, 2011). Indeed, violent video games have been found to exhibit reduced limbic activity in favor of higher activity in areas subserving cognitive control (Mathiak and Weber, 2006). More specifically, the direct comparison between the high- and low-gain NF conditions revealed a significantly higher perception of control in the NF group. Prefrontal regions and specifically the SMA are involved in self-regulation of brain activity (Hinterberger et al., 2003; Heatherton, 2011). Thereby, the target region could have a direct effect on perception of the self-regulation. The suggested changes in the VE were noticeable, thus candidates for effective NF signals.

In-game performance increased significantly with training but did not differ significantly between the groups. In general, dual tasks reduce performance by introducing attentional interferences (Pashler, 1994). The lack of difference between the NF and rc-NF groups suggests that active self-control does not further reduce task performance and hence that the dual task setup was successful. Indeed, video game has been suggested to improve dual-task performance and task-switching abilities (Strobach et al., 2012). In the participants, learning curves were observed across repetitions (Ritter and Schooler, 2001) with improvements in game performance in the VE while simultaneously trying to achieve self-regulation.

Visualization modalities for NF can provide vital information to participants about self-regulation (Kosch et al., 2016), and contextual NF has been shown to enhance reward value of successful self-regulation in target groups (Mathiak et al., 2015). Hence, perception of the NF and its integration into the VE were designed to facilitate learning during the VE NF training sessions. Participants in the NF group were able to identify and perceive changes in the respective modality (movement speed) within the VE, whereas participants in the rc-NF group were only able to correctly identify or perceive the modality changes, at slightly better than chance level while consistently rating low NF control. This is further reinforced by the significantly different subjective ratings of control between the two groups. This lack of confidence in the rc-NF group’s assessment of their effectiveness of control suggests that the NF condition was substantially different and provided an experience in which the perceived effect of NF was reinforced.

### Subjective Experience and Emotion Effects

Affect assessment with PANAS revealed no significant differences between the groups or effects of VE NF training. Accordingly, the data suggest that mood ratings were stable across scanning time. This is indeed a positive result as fMRI paradigms can often lead to boredom or loss of interest due to non-immersive stimuli, mental fatigue over time, and sometimes even physical discomfort (Sulzer et al., 2013). Consciously employing cognitive reappraisal strategies during emotional regulation has also shown to reduce subjective negative affect scores in healthy populations (Yuan et al., 2015). Conceivably, the employment of self-regulation NF strategies from NF group was more successful compared to the rc-NF group; the latter did not report a reduction in subjective negative affect after measurement. Further, the rc-NF group reported reduced subjective positive affect; this is likely caused due to the control condition, where frustration can be caused by not being able to perceive and/or control the NF modality (Sorger et al., 2019). However, this interpretation reflects results only on a descriptive level and has to be considered accordingly.

Self-Assessment Manikin rating revealed a higher arousal after the VE NF training sessions in all participants. This finding has been reported previously, indicating that video games in general tend to arouse the players (Schneider, 2004). Furthermore, it has also been suggested that arousal is closely related to presence (feeling of being immersed in a VE; Lombard and Ditton, 1997) and hence can subsequently lead to participants feeling more immersed. The SAM arousal and valence ratings yielded no significant difference. This could be due to the reduction of the SAM rating scale from a 9-point Likert scale to a 4-point Likert scale in a relatively small sample. In individual participant data, participants tended to not stray from their initial rating during the measurement. Thus, the scale seems to have little sensitivity to minor changes in arousal and valence ratings.

### Closed-Loop NF Effects on BOLD Signal

The BOLD signal data from the ROI suggest that both groups employ different regulation strategies. The average BOLD signal data from the NF group suggest that the NF group had some success in regulation of the BOLD signal throughout the gaming sessions, whereas the rc-NF group did not. On inspection of the frequency plot, the data suggest that certain frequency ranges contribute differently in their respective groups. BOLD fluctuations at different frequencies are thought to reflect separable processes (e.g., Yuen et al., 2019). In particular, slower fluctuations may be associated with introspective set shifting (Fransson, 2005) or energy metabolism (Kazemifar et al., 2017). In our study, the NF group relied more on slower signal fluctuations as well as on the high-frequency changes (0.1–0.2 Hz) in the BOLD signal as compared to the rc-NF group (see review on low frequency: Tong et al., 2019). This might reflect higher cognitive effort employed by the rc-NF group. Conceivably, the rc-NF participants tended to exercise a certain strategy for longer periods of time before noticing their failure and moving onto the next strategy. A more rigorous investigation is needed to further assess the details of the differences in the BOLD signal regulation between the groups. This task is out of the scope for this study but encourages further investigation in future work.

### Effects on Functional Connectivity

The present analysis focuses on connectivity from the feedback target at the SMA related to the navigation and problem solving task. Therefore, we studied connectivity changes with bilateral hippocampi. Even with this relatively small group, significant differences emerged in terms of a lowered connectivity in the effective NF condition compared to the rc-NF. The hippocampus contributes to mental navigation in new and recently learned environments (Hirshhorn et al., 2012) and reorientation of thoughts and forming of novel, task-related associations in problem solving (Luo and Niki, 2003). Conceivably, the segregation of the NF control from the primary task in the gaming condition reflects the learning of independent voluntary control of SMA activity during effective NF. It underpins that the VE environment can help learning of voluntary regulation of localized brain activity.

## Limitations and Outlook

Considering the complexity of the NF visualization, the in-game tasks, and the NF conditions, it becomes challenging to disentangle NF effects from the VE effects. As a first step, a separation of responses to different events may be conducted (Mathiak and Weber, 2006). Such detailed content analysis of the gaming content was out of the scope of this methodological study. Nevertheless, the prominence of difference in the low- and the high-frequency bands suggests considering events particularly in this time frame. For future studies, we suggest to introduce two additional conditions: first, a condition without VE to control for specific VE effects (Mathiak et al., 2015); such contrast could inform on the motivational increase using an interactive environment; and second, a baseline condition devoid of NF and VE to address effects of mind-wandering, which may have particularly contributed to the low-band effects (Doucet et al., 2012). Finally, the interactions between the low- and the high-frequency components need to be further clarified (Bajaj et al., 2013).

Because of the nature of the VE, one may argue that participants would have increased head motion. However, instructing and reminding the participants to be mindful of not moving while in such engagements helped in keeping the head movement to a minimum. Indeed, the estimated movement data suggest a rather limited effect of head motion (Figure 13). Accordingly, previous studies using interactive VE did not find elevated head motion parameters at the level of sessions (e.g., Mathiak and Weber, 2006). The applied NF toolbox (Koush et al., 2012) caters for minute head movements inside the scanner in real time (Mathiak and Posse, 2001).

A natural consequence of using advanced VEs is the minimum hardware and software requirements to run these paradigms. The VE needs modern and powerful graphics and central processing units to run efficiently and reliably. The paradigm also requires the use of adequate display and sound systems to deliver a more immersive experience (Audio and visual delivery systems^3^,^4^). This can restrict accessibility of such technology and reduce the probability of its use in smaller laboratories. However, with the current development of hardware, in particular with respect to graphics and 3D applications, such requirements should be addressed by standard hardware in the foreseeable future (Akçayır and Akçayır, 2017). Another aspect to take into consideration is the violent nature of the current VE; even though there is controversial evidence of the long-term effects of violent interactive media on healthy population, such a violent VE cannot be implemented in all cohorts, especially in psychopathological cohorts. However, as the backend programming does not necessitate extensive modification in future applications, it is possible to implement new non-violent VEs with a similar code using minor to moderate modifications only.

The VE, although a simplified version of a usual FPS arena, is complex to control via the MRI-compatible equipment inside the scanner. The participants also need to have experience with video-gaming, as being inexperienced leads to a much steeper learning curve. This potentially makes it harder for participants to concentrate on the NF cognitive strategies while still actively navigating in such VEs. This adds an additional confound, as only video game players are represented in the study. In future studies, the control scheme and paradigm task complexity can be optimized to lower the need of past experience with video games from the participants. The sound in the VE was unmodified from its original source; this is another avenue that can directly improve immersion if optimized for the fMRI setting.

One advantage of the VE is the real-time logging of data from the gaming sessions during the fMRI measurement. This significantly reduces the time investment and personnel overhead in producing data for analysis. However, this pipeline is still inefficient. In the current state, the data need to be imported into different software where it is rearranged and reformatted so that it may be used for analysis (Ramadan et al., 2019). This process needs to be automated for future work, and possibly the export of the log file needs to be optimized for multiple output formats.

## Conclusion

Our aim in this project was to develop and implement a novel rt-fMRI–based NF training paradigm, taking into account new technological advances that allow us to integrate complex stimuli into a visually updated and engaging VE. We built up on and developed a shooting game in which the dynamic change in the VE was the feedback variable of this BCI. This novel method of presenting NF implicitly within the task paradigm was implemented into an fMRI setting. We designed a study to assess the feasibility of the BCI in creating a cost-effective and immersive VE for the NF training. The experimental manipulation showed that participants perceived and reported control over the presented NF stimulus. The participants were engaged within the VE, improving their performance metrics over time while simultaneously employing NF strategies. The NF group tended to finish the measurement reporting improved mood. The study found group differences in BOLD responses and connectivity due to the NF.

## Data Availability Statement

All software, data and similar resources used in the experiment are available on request from the authors.

## Ethics Statement

The studies involving human participants were reviewed and approved by Independent Ethics Committee of the University Hospital RWTH Aachen. The patients/participants provided their written informed consent to participate in this study.

## Author Contributions

HB, MKl, JZ, MZ, RG, and KM contributed to the conception and design of the study. HB, SR, and MZ contributed in programming/modifying the neurofeedback toolbox for the study. HB recruited and measured participants with assistance from LR. HB performed the data analysis with guidance from JZ, MKe, ER, and KM. HB programmed/modified the virtual environment paradigm and wrote the manuscript. All the authors contributed to manuscript revision.

## Conflict of Interest

The authors declare that the research was conducted in the absence of any commercial or financial relationships that could be construed as a potential conflict of interest.

## Footnotes

1 www.fil.ion.ucl.ac.uk/spm/

2 https://www.nitrc.org/projects/wfu_pickatlas

3 https://www.crsltd.com/tools-for-functional-imaging/

4 https://nordicneurolab.com/nordic-fmri-solution/
